# Determinants in Tailoring Antidiabetic Therapies: A Personalized Approach

**DOI:** 10.1055/s-0041-1741109

**Published:** 2022-06-13

**Authors:** Aliya A. Rizvi, Mohammad Abbas, Sushma Verma, Shrikant Verma, Almas Khan, Syed T. Raza, Farzana Mahdi

**Affiliations:** 1 Department of Personalized and Molecular Medicine, Era University, Lucknow, Uttar Pradesh, India; 2 Department of Biochemistry, Era University, Lucknow Medical College and Hospital, Lucknow, Uttar Pradesh, India

**Keywords:** diabetes, precision medicine, pharmacogenomics, drug response

## Abstract

Diabetes has become a pandemic as the number of diabetic people continues to rise globally. Being a heterogeneous disease, it has different manifestations and associated complications in different individuals like diabetic nephropathy, neuropathy, retinopathy, and others. With the advent of science and technology, this era desperately requires increasing the pace of embracing precision medicine and tailoring of drug treatment based on the genetic composition of individuals. It has been previously established that response to antidiabetic drugs, like biguanides, sulfonylureas, dipeptidyl peptidase-4 (DPP-4) inhibitors, glucagon-like peptide 1 (GLP-1) agonists, and others, depending on variations in their transporter genes, metabolizing genes, genes involved in their action, etc *.* Responsiveness of these drugs also relies on epigenetic factors, including histone modifications, miRNAs, and DNA methylation, as well as environmental factors and the lifestyle of an individual. For precision medicine to make its way into clinical procedures and come into execution, all these factors must be reckoned with. This review provides an insight into several factors oscillating around the idea of precision medicine in type-2 diabetes mellitus.

## Introduction

Diabetes refers to a hyperglycemic condition that may be caused either by a deficiency of insulin or defects in its action or both. Hyperglycemia that results from defects in insulin action, also referred to as insulin resistance, is called type-2 diabetes mellitus (T2DM) or noninsulin-dependent diabetes mellitus (NIDDM). 1 According to the data provided by the International Diabetes Federation (IDF), there are approximately 74,194.7 people aged between 20 and 79 years in India who had diabetes as of 2021. 2 Obesity is common in individuals suffering from T2DM and is believed to cause some degree of insulin resistance. 3 A major cause of this metabolic disorder is a sedentary lifestyle and unhealthy eating habits. The early stages of this metabolic disorder can be controlled by proper diet and exercise, whereas later stages require treatment with antidiabetic drugs. 3 4

Physicians may prescribe these drugs alone (monotherapy) or in combination (combination therapy) according to the predisposition of the disease and in a set order. If unresponsiveness is observed with first-line therapy or toxicity occurs treatment shifts to subsequent lines of treatment according to the guidelines provided by the World Health Organization (WHO). 5 Commercially available antidiabetic drugs include metformin, sulfonylureas (SU), glucagon-like peptide 1 (GLP-1) agonists, dipeptidyl peptidase-4 (DPP-4i) inhibitors, and others *.* There are several genes whose polymorphisms affect an individual's response to these antidiabetic drugs including drug transporter genes like organic cation transporters ( *OCTs* ) viz., *OCT1, OCT2,* *OCT3* , multidrug and toxin extrusion transporter 1 ( *MATE1* ), drug-metabolizing genes like cytochrome P450 genes ( *CYP2C8* , *CYP2C9* ), and genes involved in drug action. The effect of genes on drug response is studied under “pharmacogenetics” which is established on the idea that an individual's genetic makeup and the structure, concentration and configuration of the proteins thus expressed, influences drug response in many ways. 6 7 The structure and configuration of a protein depend on the genetic information and any variation in genes might alter the amino acid composition of the protein, folding of the protein, and structure of its active site. If the variation is present in the promoter region or its regulator genes, it will either upregulate its expression increasing the concentration or vice versa. Such variations in genes effectuate individuals to respond differently to certain drugs.

Epigenetic factors, like microRNAs, DNA methylation, and histone modifications, have also been studied to influence susceptibility to T2DM and response to antidiabetic drugs. Apart from genetic and epigenetic variations, there are other factors including environment, lifestyle, diet, and others, which determine susceptibility and drug response. These determinants concerning customization of antidiabetic therapies have been discussed in detail in this review.

## An Insight into Precision Medicine

**Fig. 1:**
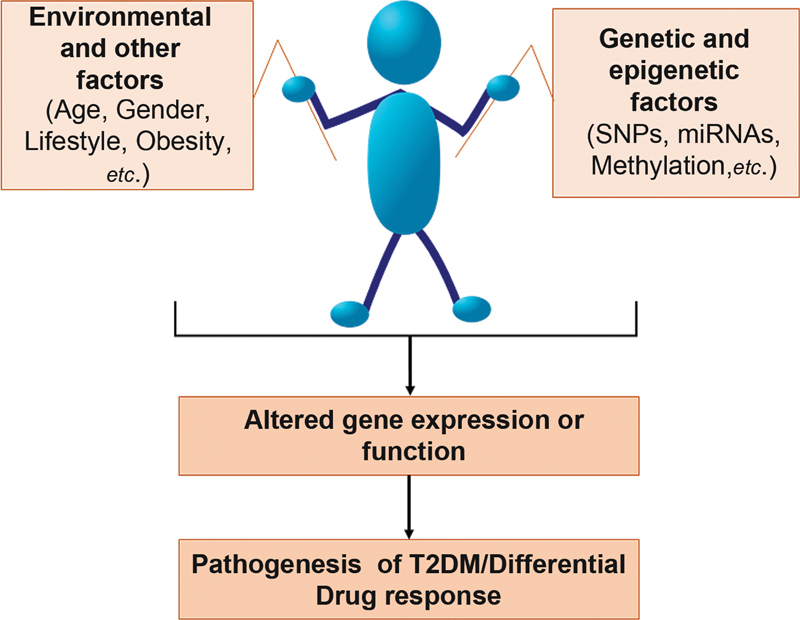
Determinants of disease risk and pathogenesis as well as response to drug therapies. SNPs, single nucleotide polymorphisms; T2DM, type-2 diabetes mellitus.

“Pharmacogenetics,” a term coined by Vogel refers to the study of people's genetic variations which affect the way they respond to drugs. Differences in their genes affect the expression, structure, and configuration of the related proteins and this becomes the reason for different people responding differently to identical drug therapies. 6 7 Several other factors, such as environmental factors, dietary habits, physical activity, age, sex, ethnicity, and other comorbidities, also cause a variable response to the same drug. 8 Considering this variable response, it becomes necessary to introduce “precision medicine.” Precision medicine refers to the use of medication, taking into account all the factors which can affect his response to therapeutics including his genetic makeup, lifestyle, and environmental factors because these, all together, are responsible for efficacy, neutrality, or toxicity of a particular drug. 9 It has been shown that although metformin is used as first-line therapy for T2DM but some people respond positively whereas, in some, it causes adverse effects due to variations in related genes, and thus they require the use of alternate drugs. This is the reason that precision medicine is getting a lot of attention at present concerning disorders like T2DM, cancer, hypertension, and others *.* International HapMap project, which is an off-shoot of the Human Genome Project, aims to identify and catalog single nucleotide polymorphisms (or SNPs) which are sequences of genetic variants in diverse populations. 10 The SNPs related to a particular disease can thus be identified, correlated with other factors (environment, lifestyle, etc.), and accordingly precision medicine can be made available based on individual's genetic information ( Fig. 1 ).

## Genetic Markers for Type-2 Diabetes Mellitus

Genetic variations may be protective against a particular disease or they can increase a person's susceptibility for a particular disease. Genetic markers for a disease are the genetic sequences or variations which may lead to its onset, progression, or severity. Some genetic markers also determine whether or not an individual will respond to a particular drug. By far, there are more than a hundred risk alleles that have been linked to T2DM in genome-wide association studies (GWAS). 11 These alleles may influence β-cell function, insulin resistance, drug metabolization, drug transport, etc *.* Since T2DM is a heterogeneous and polygenic disease, identifying the most potent genetic biomarkers and applying the precision medicine approach is more difficult in the case of T2DM as compared with monogenic forms of diabetes. 12

A study performed on 37,000 individuals identified 10 loci that reduced β-cell function and 3 that were linked to insulin sensitivity. The loci that were associated with reduced β-cell function were melatonin receptor 1B ( *MTNR1B* ), solute carrier family 30 member 8 ( *SLC30A8* ), thyroid adenoma–associated protein ( *THADA* ), transcription factor 7-like 2 *TCF7L2* , potassium voltage–gated channel subfamily Q member 1 ( *KCNQ1* ), calcium/calmodulin-dependent protein kinase 1D ( *CAMK1D* ), etc *.* Three loci that were associated with insulin sensitivity were peroxisome proliferator-activated protein gamma ( *PPARG* ), fat mass and obesity-associated gene ( *FTO* ), and Kruppel-like factor 14 ( *KLF14* ). 13 Metabolites, like glycine, lysophosphatidylcholine (LPC), and acetyl carnitine, have been previously known to have disturbed levels in patients with impaired glucose tolerance (IGT) 14 and an in silico study showed an association between these metabolites and T2DM-associated genes, that is, insulin-like growth factor 1 ( *IGF1* ), insulin receptor substrate 1 ( *IRS1* ), insulin-degrading enzyme ( *IDE* ), *TCF7L2* , *PPARG* , and others *.* These genes were found to be efficient prediabetic markers in patients from United Arab Emirates (UAE). 14 Among all the genetic markers, *TCF7L2* is the largest susceptibility for T2DM and two of its variations, that is, rs7903746 and rs12255372, are the strongest and most efficient biomarkers. 15 16 17 Some drug transporter genes like organic cation transporters ( *OCTs* ) have also been associated with increased T2DM susceptibility in several studies. 18

## Genetic and Epigenetic Determinants of Response to Type-2 Diabetes Mellitus Therapeutics

Metformin, an oral antidiabetic drug, is a biguanide and is used as the first-line therapy for T2DM. Second-line treatment involves use the of SU, whereas insulin or DPP-4 inhibitors, sodium-glucose cotransporter 2 (SGLT-2) inhibitors, or a thiazolidinediones form the third-line treatment as recommended by WHO. 5 As previously discussed, the response to these drugs depends on alterations in the associated genes which include their transporters, metabolizers, and those involved in their action as well as their regulators. Genes, like *OCT1, OCT2, OCT3, MATE1, MATE2,* and others, are related to metformin response while cytochrome P450 (CYP) genes like *CYP2C9* , *TCF7L2* , potassium inwardly rectifying channel, subfamily J, member 11 ( *KCNJ11* ), etc *.* , mainly influence SU response and response to DPP-4 inhibitors is altered by genes like *DPP-4* , *GLP-1* receptor ( *GLP1R* ), *KCNQ1* , *KCNJ11* , cyclin-dependent kinase 5 regulatory–associated protein 1-like-1 ( *CDKAL1* ), and others. 19 Similarly, response to a novel class of antidiabetic drugs, that is, GLP-1 agonists is affected by *TCF7L2* and the Wolframin endoplasmic reticulum transmembrane glycoprotein ( *WFS1* ), etc. 20 21

### Genetic Determinants of Metformin Response

Metformin is a derivative of guanidine, first discovered in the 1920s in the Galega officinalis (French lilac) extracts. 22 It does not require metabolizing enzymes for its action. 23 The main site of action for metformin is the liver where it is taken up by its transporter genes like *OCT1* and then by acting via adenosine monophosphate (AMP)-associated protein kinase ( *AMPK* )-dependent pathway or AMPK-independent pathway, it alters ADP/AMP ratio thereby activating *AMPK* and inhibition of mitochondrial respiratory chain. 23 The hepatic uptake and renal transport of metformin is primarily regulated by its transporter OCT1, and any alteration in this gene or protein thus expressed, directly affects metformin efficacy. It is coded by a highly polymorphic gene, *SLC22A1* , and these polymorphisms cause variable transport function. 24 25 There are six common SNPs of *OCT1* highly studied in association with T2DM. These are Met408Val (rs628031), Met420del (rs72552763), Phr160Leu (rs6383369), Pro341Leu (rs2282143), Gly401Ser (rs34130495), and an intronic SNP, rs622342. Concerning metformin response, Met408Val (rs628031) is the one massively studied SNP of *OCT1* where AA genotype showed a significant reduction in HbA1c levels ( *p* < 0.02) compared with those having heterozygous AG genotype in Han Chinese population after metformin therapy. 26 27 The same variant has also been shown to counter the effect of Met420del variant where deletion of 3bp (GAT) reduces transport function of *OCT1* . 24 28 High association of intronic variant rs622342 with efficacy of metformin has been shown in which the major allele A had 5.6 fold higher chances to respond to metformin 29 and the minor allele C was associated with approximately 0.28% lower decrease in HbA1c levels. 30 Slight reduction in function of *OCT1* was observed with Pro341Leu and Phe160Leu. 31 32

**Fig. 2:**
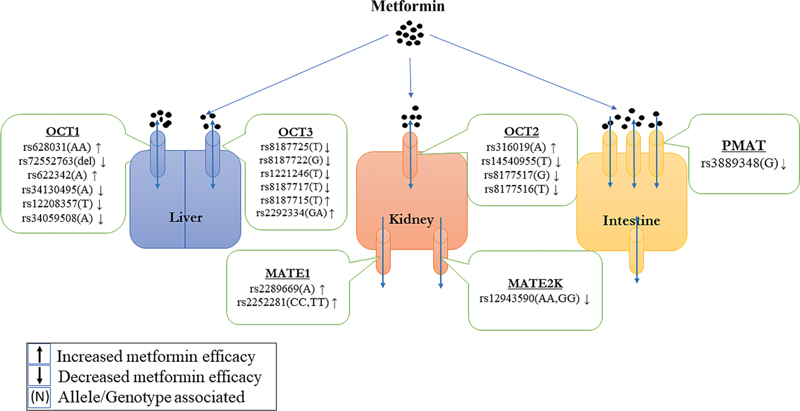
Variations in transporter genes influencing metformin action in different organs. Given in brackets are the alleles/genotypes of the SNP that are associated with metformin response. (OCT1, 2, and 3, organic cation transporters 1, 2, and 3; MATE 1 and 2, multidrug and toxin extrusion transporters 1 and 2; PMAT, plasma membrane monoamine transporter.

Five SNPs suggested for pharmacogenetic studies by the International Transporter Consortium are rs34130495 (Gly401Ser), rs12208357 (Arg61Cys), rs34059508 (Gly465Arg), rs55918055 (Cys88Arg), and rs72552763 (Met420del). Minor alleles of all of these SNPs, that is, A, T, A, C, respectively, and deletion in case of Met420del, reduce the expression of *OCT1* gene and thus metformin effectiveness. 33 Renal transport of metformin is mainly associated with *OCT2* , *MATE1* , and *MATE2* genes. *OCT2* , which is majorly expressed in renal tubule cells, regulates entry of metformin into renal tubular cells. 34 Minor allele of rs316019 of *OCT2* , that is, allele A, has been known to cause elevated plasma levels and reduced renal clearance of metformin due to decreased function of the transporter gene. 35 Another study performed on African Americans, European Americans, Asians, and Mexicans showed better uptake of metformin with the same variant. 36 Other variants of *OCT2* like rs14540955, rs8177517, rs8177516, rs201919874, and rs8177507 had their variant alleles viz., T, G, T, T, and A, respectively, associated with reduced transporter activity. 18 *OCT3* is another major metformin transporter that is involved in its absorption and tissue distribution. 37 38 39 Variant alleles of several exonic variants of OCT3 have been shown to reduce metformin uptake, viz., T alleles of rs8187725, rs1221246, and rs8187717 and G allele of rs8187722. 18 40 41 Two other variants, that is, rs8187715 and rs2292334, studied separately showed better metformin efficacy with T allele of rs8187715 associated with increased uptake activity of the transporter and heterozygous variant (GA) of rs229334 showed high plasma concentration and lower elimination of the drug. 40 41 42 43 *MATE1* and *MATE2* genes are associated with renal elimination of metformin. 44 Minor allele, that is, A allele of rs2289669 and homozygous genotypes (CC and TT) of rs2252281 of *MATE1* both showed better response with metformin monotherapy whereas homozygous genotypes (AA and GG) of rs12943590 (−130G > A) of *MATE2* conferred to reduced response by increasing renal clearance of metformin. 45 46 47 48 G allele of a polymorphism in plasma membrane monoamine transporter ( *PMAT* ) gene, i.e., rs3889348, caused gastrointestinal intolerance to metformin in several populations 49 ( Fig. 2 ).

### Genetic Determinants of Sulfonylureas Response

**Fig. 3:**
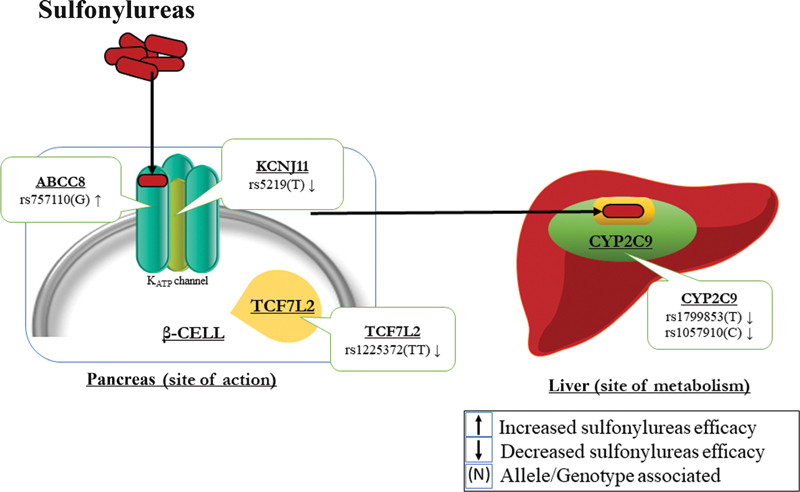
Polymorphisms determining fate of sulfonylureas. Given in brackets are the alleles/genotypes of the SNPs that are associated with sulfonylureas response. ABCC8, ATP-binding cassette, subfamily C, member 8; CYP2C9, cytochrome P450; KCNJ11, potassium inwardly rectifying channel, subfamily J, member 11; TCF7L2, transcription factor 7-like2..

If metformin alone fails to achieve desired reduction in HbA1c levels, it is recommended to combine it with other drugs like SU, DPP-4 inhibitors, or sodium-glucose cotransporter 2 (SGLT2) inhibitors as the first choice or TZDs, glinides, or GLP1RA as second choice as directed by the Indian Council of Medical research and WHO. 5 50 SU works by activating potassium channels stimulating the release of insulin from β-cells of pancreas. 51 Sulfonylurea receptor 1 (SUR1) presents on ATP-sensitive potassium channels (K- _ATP_ ) that is encoded by the ATP-binding cassette, subfamily C, and member 8 ( *ABCC8* ), whereas potassium inwardly rectifying channel, subfamily J, member 11 ( *KCNJ11* ) encodes pore subunit of K _ATP_ channel, Kir6.2. 52 rs5219 of *KCNJ11* is the most common SNP of this gene which is associated with improved response to SU in Chinese population; however, other studies have associated T allele of this variant with decreased expression of the subunit and increased risk of SU failure. 53 54 Improved response to SU has been found in carriers of G allele of nonsynonymous variant, rs757110 of *ABCC8* gene. 54 55 SUs are dependent on drug metabolizing enzymes coded by genes, like cytochrome P450 genes, *CYP2C9* , *CYP2C8* , and others, for their metabolism. The main enzyme that metabolizes SU is *CYP2C9* . 56 Variant alleles of two variations in this gene, that is, T allele of rs1799853 ( CYP2C9*2 ) and C allele of rs1057910 ( CYP2C9*3 ) are associated with poor response to SU since the protein expressed from these variants has reduced function. 56 57 58 59 As for *TCF7L2* , apart from increasing the risk of T2DM, this gene also influences SU response since it directly affects β-cell function. It was found that TT genotype of rs1225372 conferred to failure to attainment of lower level of HbA1c (i.e., 7% or less) as compared with GG genotype ( *p* = 0.006). 60 Polymorphisms of two other genes which are T2DM susceptibility genes, that is, insulin receptor substrate-1 ( *IRS-1* ) and nitric oxide synthase 1 adaptor protein ( *NOS1AP* ), viz., rs1801278 (Gly972Arg) and rs10494366, respectively, also contribute to SU response. While AA and GA genotypes of rs1801278 of *IRS-1* were associated with increased failure of SU response, GT, and GG genotypes of rs10494366 of *NOS1AP* correlated with increased mortality rate in patients on SU 61 62 ( Fig. 3 ).

### Genetic Determinants of Dipeptidyl Peptidase-4 Inhibitors

DPP-4 enzyme degrades the hormones GLP-1 and glucose-dependent insulinotropic polypeptide (GIP). 63 DPP-4 inhibitors are the class of antidiabetic drugs that prevent the degradation of GLP-1 and GIP by DPP-4 thus decreasing the levels of glucagon and improving responsiveness of β-cells to increased glucose concentrations. 64 These incretin hormones have a very short half-life and play a role in glucose-dependent insulin secretion, suppression of glucagon secretion, etc. 65 66 There are several genes whose variations alter the responsiveness to DPP-4 inhibitors in individuals with T2DM, viz., *DPP-4* , GLP-1 receptor ( *GLP1R* ), *KCNQ1* , *KCNJ11* , *CDKAL1* , and others. 67 DPP-4 is the substrate for DPP-4 inhibitors that bind to it so that inactivation of GLP-1 can be prevented. 68 Efficacy of a DPP-4 inhibitor, sitagliptin, was found to be lower in individuals having TT genotype of rs2909451 and GG genotype of rs4664443 of the gene for *DPP-4* enzyme. 69 Studies have reported that homozygotes for A allele rs6923761 of *GLP1R* observed lower glucose-lowering effect of DPP-4 inhibitors, 70 71 whereas A allele of rs3765467 was associated with better response to DPP-4 inhibitor therapy. 71 Similarly, SNPs of *KCNQ1* gene, which helps in GIP and GLP-1 secretion in intestines, 72 influence DPP-4 therapy whereas G allele of rs163184 conferred to lower response. 73 CC genotype of a polymorphism rs2285676 in *KCNJ11* gene, which stimulates insulin secretion from β-cells, 74 showed two-fold higher probability of response to DPP-4 inhibitors. 75 Two variants of *CDKAL1* , namely, rs7754840 (C > G) and rs756992 (A > G) had greater decrease in HbA _1c_ with at least one variant allele, that is, G and A allele, respectively, in comparison to those having common allele. 76

### Genetic Determinants of Glucagon-Like Peptide-1 Agonists Response

Recent guidelines issued by American Diabetes Association recommend GLP1 agonists as second line treatment for diabetic patients with cardiovascular disease or SGLT2 inhibitors if there are established comorbidities like chronic kidney disease, heart failure, and others, whereas SU, DPP-4 inhibitors and TZDs are recommended as fourth line therapies after combination therapies and GLP-1 agonists. 77 GLP1 agonists are a new class of antidiabetic medication. GLP1, as discussed earlier, is an incretin hormone that decrease the levels of glucagon, increases glucose-dependent insulin secretion. 78 There are a few genes whose polymorphisms have studied to alter response to exogenous GLP-1 (GLP-1 agonists) like *TCF7L2* , *WSF1* , and *GLP1R* . Risk alleles of *TCF7L2* and *WFS1* variants, that is, rs7903146 (C > T) and rs10010131, respectively, were both significantly associated with reduced responsiveness to GLP-1 agonists, 20 21 whereas variants of *GLP1R* , that is, rs3765467, rs761386, and rs587654, studied in Japanese population were not significantly associated with response to exogenous GLP-1. 79

## Role of Epigenetics

Epigenetic modifications are heritable changes that affect the expression of genes which include DNA methylation and histone modification (methylation, acetylation, SUMOylation, etc.). Most of these modifications correlate with repression of transcription like DNA and histone methylation, whereas some associate with upregulation of gene expression, for example, histone acetylation. 80 MicroRNAs, or miRNAs, are another important epigenetic factor which regulate expression posttranscriptionally. Upon binding to the target mRNAs on their 3′-UTR (untranslated region), they destabilize mRNA, thereby repressing protein expression. 81 Although all the epigenetic factors are important, there are fewer studies associating miRNAs and histone modifications with T2DM susceptibility, pathogenesis, and drug response as compared with DNA methylation which has been extensively studied. 82

DNA methylation is the transfer of methyl group to cytosine ring catalyzed by DNA methyltransferases (DNMT) occurring mainly in those cytosine rich regions which are followed by a guanine. These regions are known as “CpG islands” which are usually unmethylated and more frequently found in the promoter region. DNA methylation is a method of controlling gene expression which mainly locks the genes in “off” position known as gene silencing. 83 84 85 Lower expression of transporter genes of metformin, that is, *OCT1, OCT3* , and *MATE1* , as a result of DNA hypermethylation was observed in T2DM patients which reduced metformin uptake and transport. 33 36 37 In addition to this, direct diminution of methylation of these transporter genes was observed with metformin. 86 A study performed on Greek patients to investigate the relationship between promoter methylation of KCNJ11 and ABCC8 genes and hypoglycemia linked with SU concluded that methylation of only ABCC8 gene prevented the risk of SU-linked hypoglycemia. 87 Yet another study associated hypermethylation of promoter of vault RNA 2–1 ( *VTRNA2–1* ) with better responsiveness to GLP-1 analogs. Also, A allele of a polymorphism in the same gene, that is, rs2346018, correlated with hypomethylation and thus reduced responsiveness to GLP-1 analogs. 88

miRNAs have a role in posttranscriptional regulation of expression. These are 20 to 30 nucleotides-long noncoding RNAs which bind to 3′-UTR of the target primary transcript causing its destabilization and repression of translation. 81 89 Several miRNAs have been associated with T2DM susceptibility, like miR-375, miR-146a, and others, which were overexpressed in T2DM patients. 89 Apart from imparting susceptibility, some miRNAs have also been shown to be involved in action few antidiabetic drugs. One example of this is DPP-4 inhibitor linagliptin which alleviates kidney fibrosis by reversing the DDP-4-induced repression of miRNA29s. 90 A study found that expression of *KCNJ11* was influenced by rs60432575 G > A of *MIR4532* , hence causing reduction in sulfonylureas-mediated insulin secretion. 91

## Other Factors Affecting Drug Response

Apart from genetic and epigenetic variations, there are other factors that affect how an individual would respond to a particular drug. These factors include ethnicity, age, gender, lifestyle, obesity, and others *.* It has been widely accepted that obesity has a significant role in causing insulin resistance. Also, increasing age elevates the risk of T2DM susceptibility. Lifestyle factors including type and amount of stress, eating habits like intake of saturated fats, increasing weight, decreased amount of dietary fiber, decreased physical activity, smoking habits, alcohol intake, etc., alleviate a person's risk of developing T2DM and can also interfere with drug response. 92 With respect to exercise, although it maintains blood-glucose level by increasing translocation of glucose transporter type 4 (GLUT4) and eventual uptake of glucose in active muscles, 93 it has been observed that glucagon levels were the highest when exercise was combined with metformin therapy. 94 In case of meglitinides and SU, there remains risk of hypoglycemia if combined with exercise and it is the highest with SU and insulin. When metformin was combined with exercise, hypoglycemia was found to occur only in individuals consuming excessive alcohol or those suffering from severe hepatic insufficiency. 95 96 Therefore, it can be concluded that regular exercise might decrease T2DM risk but with respect to antidiabetic therapies, care must be taken, so that no adverse effects occur. Also, if some adjustments are made with dosage and exercise, the risk of hypoglycemia associated with some medications can be avoided. 97

## Conclusion and Future Prospects

T2DM, with a worldwide prevalence as high as 6.28%, 98 needs a hurried transition in its management from disease centric to explicitly patient centric. There are numerous studies already done and still going on concerning genetic factors for susceptibility to T2DM and personalizing diabetes treatment yet the pace of execution is excruciatingly slow. Tailoring drug treatment based on genetic profiles of can be procured by applying the knowledge obtained so far in modifying guidelines for diabetes care and management. As described in this review, environment, lifestyle (smoking habits, alcohol consumption, body weight, physical activity, etc.), genetic (SNPs, mutations, etc.), and epigenetic factors (DNA methylation, histone modifications, and miRNAs), and others influence drug response in several ways. These factors are variable in different individuals and the ultimate repercussion of these variations is on drug response or susceptibility and pathogenesis of the disease. Although there are limitations like T2DM being a highly heterogeneous disease, difficulty in implementing genetics-based care and treatment in some populations, etc *.* , these can be subjugated by more studies performed on different populations and larger cohorts. With cost-effective genotyping assays, genetics-based health would be easier to embrace. Since it is more feasible to control T2DM in the early stages, there is also a dire need to screen all those at the risk of developing T2DM or going toward severity of the disease. Also, before the commencement of treatment, patients must be genotyped and the results must be correlated with lifestyle, clinical, environmental, and epigenetic aspects to differentiate them into responders and nonresponders, so that appropriate drugs and doses could be prescribed preventing any toxicity of antidiabetic drugs.
